# Synergistic effect of reduced polypeptide micelle for co-delivery of doxorubicin and TRAIL against drug-resistance in breast cancer

**DOI:** 10.18632/oncotarget.11451

**Published:** 2016-08-20

**Authors:** Chuling Hu, Fenfen Gu, Zongguang Tai, Chong Yao, Chunai Gong, Qingming Xia, Yuan Gao, Shen Gao

**Affiliations:** ^1^ Department of Pharmaceutics, Changhai Hospital, Second Military Medical University, Shanghai 200433, China

**Keywords:** polyarginine, lipoic acid, reduction-sensitive, drug resistance, cancer therapy

## Abstract

Cationic peptides as a non-viral gene vector have become a hotspot of research because of their high transfection efficcacy and safety. Based on our previous study, we synthesized a cationic reduction-responsive vector based on disulfide cross-linked L-arginine, L-histidine and lipoic acid (LHRss) as the co-carrier of both doxorubicin (DOX) and the necrosis factor-related apoptosis-inducing ligand (pTRAIL). The LHRss/DOX/TRAIL construct has reduction-sensitive behavior and an enhanced endosomal escape ability to increase the cytotoxicity of DOX and the transfection efficiency. Further, the LHRss/DOX/TRAIL construct increased the accumulation of DOX and promoted the expression of pTRAIL, thus increasing cellular apoptosis by 83.7% in MCF-7/ADR cells. In addition, the *in vivo* biodistribution results showed that the LHRss/DOX/TRAIL construct could target tumors well. The *in vivo* anti-tumor effect study demonstrated that the LHRss/DOX/TRAIL construct inhibited tumor growth markedly, with a tumor inhibitory rate of 94.0%. The co-delivery system showed a significant synergistic anti-tumor effect. The LHRss/DOX/TRAIL construct may prove to be a promising co-delivery vector for the effective treatment of drug resistant breast cancer.

## INTRODUCTION

Multidrug resistance (MDR) often leads to failure of breast cancer chemotherapy because MDR may increase cell surface drug-pump protein efflux, inhibit cancer cell apoptosis, decrease drug influx, and alter cell cycle regulation [1, 2]. Tumor cells are characterized by a strong proliferative ability, but the most important feature of tumor cells is their loss of the ability to undergo spontaneous apoptosis. Thus, the study of apoptosis in tumors is one of the more promising breakthrough points in tumor chemotherapy [3]. It was reported that tumor necrosis factors (TNF) such as TNF-α can increase tumor cell apoptosis and enhance the efficacy of chemotherapy drugs [4]. TNF-related apoptosis-inducing ligand (TRAIL) has recently been identified as a protein of interest because of its remarkable ability to induce rapid apoptosis of tumor cells without affecting most normal cells. TRAIL induces apoptosis by interacting with death receptor 4 (DR4) and death receptor 5 (DR5), leading to the formation of the death-inducing signaling complex (DISC), which binds caspase-8 [4, 5]. The recruitment of caspase-8 to the DISC activates its proteolytic properties, thus initiating a cascade of protease activation involving enzymes such as caspase-3, which promotes the subsequent cleavage of death substrates and results in apoptosis. Herein, co-delivery TRAIL and chemotherapeutic drugs may increase the synergistic effects of drug efficacy and prohibit the MDR of chemotherapy. However there are challenges of the transport of chemotherapeutic drugs and gene drugs in the co-delivery systems such as ineffective cellular transfection, instability *in vivo* of gene drug, as well as non specific target effect of drugs [6, 7].

Effective delivery systems can cross various barriers *in vivo* and intracellularly deliver drugs to the target tumor cells and cause an antitumor effect [8, 9]. Therefore, these delivery systems should have multiple functions such as long-term stability, target specificity, and the ability to enhance endosomal escape [10]. The synthesis of functional units, a technology using supramolecular groups to assemble multiple functional units into a combination, is an effective method to obtain multi-functional carriers. The ordered structure of self-assembly with the appropriate nano-size and a controllable structure that can cross a series of barriers, such as the cell membrane, the endosomal membrane, and the nuclear membrane, has great potential prospect [11].

In this study, we developed a nanosystem based on disulfide cross-linked lipoic acid modified polyarginine peptide and histidine (LHRss) for co-delivery of the chemotherapeutic drug doxorubicin (DOX) and TRAIL to MDR breast cancer cells. Polyarginine, as a cell penetrating peptide, was used as an intracellular delivery vector, knowing that it can effectively compress DNA by enriching the positive charges in the DNA [12, 13], and histidine can help nanomicelle endosomal escape through the proton sponge effect [14, 15]. Moreover, the intermolecular disulfide bonds of lipoic acid can avoid instability by self-assembling into a nanomicelle *in vivo* that is cracked in the glutathione-reducing conditions in tumor cells, releasing the drug and avoiding the failure of effective drug release in cells [16, 17]. The LHRss/DOX/TRAIL construct was assembled into a nanomicelle structure with DOX encapsulated in the hudrophobic core and pTRAIL condensed on the hudrophilic layer. We hypothesized that the LHRss/DOX/TRAIL construct could effectively enhance the cytotoxicity of the drug, promote the apoptotic response, providing a targeted delivery and enhanced effect of DOX and TRAIL to drug-resistant breast cancer cells (Figure 9). For this purpose, cellular uptake, *in vitro* transfection efficiency, cytotoxicity, cellular apoptosis rate, *in vivo* distribution as well as anti-tumor effect of the LHRss/DOX/TRAIL construct were investigated in a drug-resistant breast cancer xenograft nude mice model.

## RESULTS AND DISCUSSION

### Characterization of LHRss

**Figure 1:**
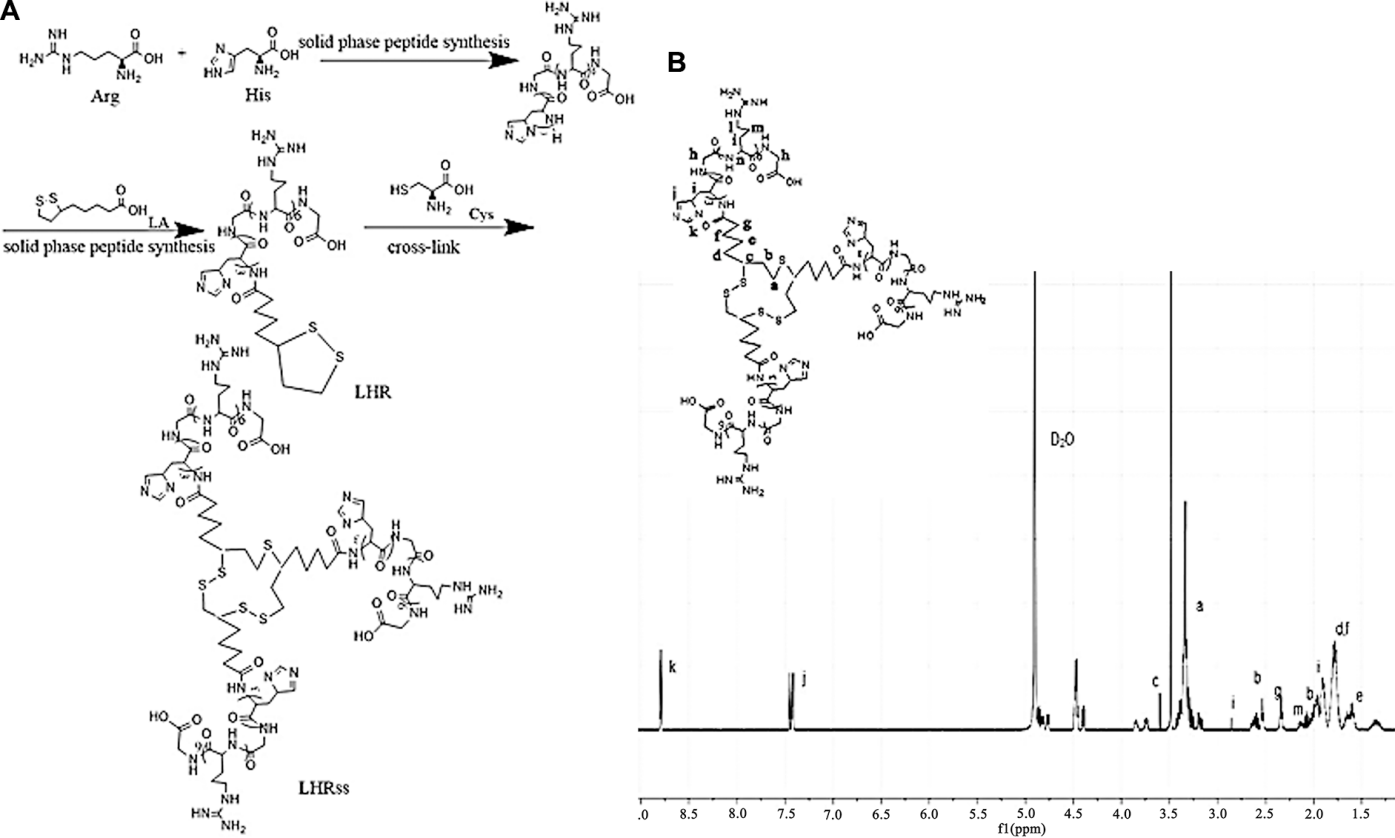
Synthesis and ^1^H-NMR determination of LHRss (A) Synthesis of LHRss and (B) ^1^H-NMR spectra of LHRss in D_2_O at 600 MHz Abbreviations: Arg, arginine; His, histidine; LA, L-lipoic acid; cys, cysteine; LHR, lipoic acid modified polyarginine peptide and histidine (LHRss); LHRss, disulfide cross-linked lipoic modified with polyarginine peptide and histidine.

LHR was synthesized using F-moc-SPPS, and cysteine was used as a cross-linking agent to cross-link an intermolecular disulfide bond of lipoic acid to obtain the appropriate molecular weight for further research. At the same time, methanol was used as a solvent. After completion of the reaction, the solvent was removed by N_2_ drying, and the remaining cysteine was neutralized by NaOH. The peptide LHR was synthesized with > 95% purity and a precise molecular weight of 1554.88 Da. The mass-average molecular weights (Mw) of the polymers are summarized in Table 1. The Mw of the LHRss was 22.9 kDa when the molar ratio of cysteine to LHR was 10%, and the Mw of the LHRss was significantly enhanced compared to that of the original monomer LHR (1544.88 Da). The formation of LHRss was verified by ^1^H-NMR (Figure 1B). Figure 1B shows the ^1^H NMR results of LHRss, indicating that δ3.59 ppm (signal c) belonged to the methyne of the lipoic acid, and δ2.34 ppm (signal g) belonged to the hydrogen on the methylene group of the carbonyl group. δ3.48 ppm, 2.58 ppm and δ1.36~1.59 ppm were from the proton of lipoic acid moiety (signals a, b, e, d and f, respectively). The peak at δ1.79 ppm (signal i) and the peak at δ2.04 ppm were attributed to −CH2−close to the tertiary carbon in arginine and histidine (signal m). Signals d (δ1.54 ppm) and h (δ3.19 ppm) were attributed to the rest of −CH2−in arginine. The peaks at δ4.23−4.62 ppm(signal n) were due to protons of tertiary carbon in the polymer. The peak at δ7.17 ppm (signal j) and δ8.54 ppm (signal k) were from protons of imidazole in histidine.

**Table 1:** The synthetic conditions and the corresponding molecular weight of LHRss

Polymers	LHR(mg)	Cysteine(mg)	Feed ratio(%)^a^	Mw(kDa)^b^
LHR	−	−	−	1.555
LHRss	50	0.39	10.0	22.9

Notes:

a Molar ratio of cysteine to LHR.

b Data obtained by gel permeation chromatography.

### Characterization of LHRss/DOX/pGL3 complexes

**Figure 2:**
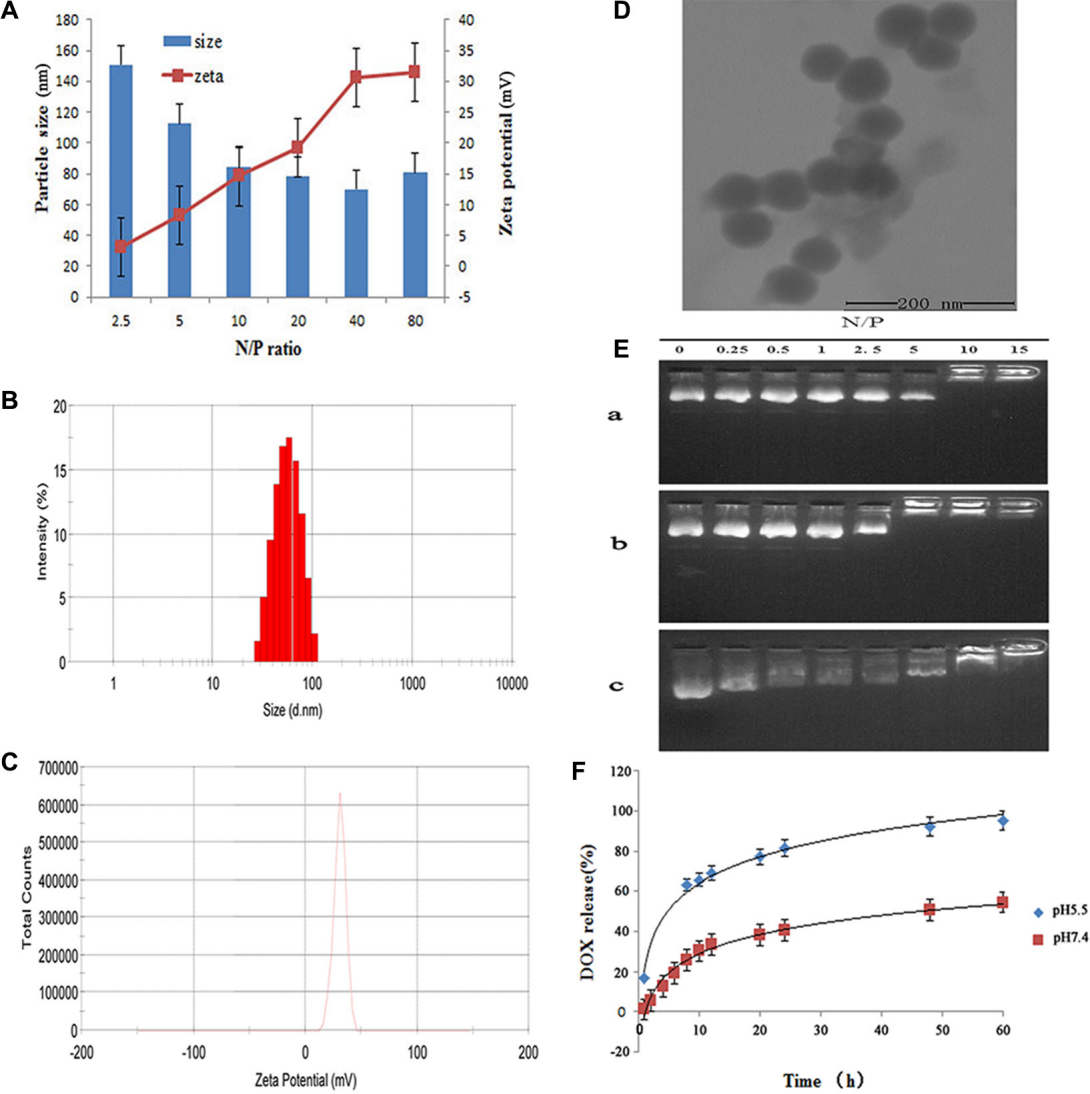
Characterization ofLHRss/DOX/TRAIL (**A**) Particle size and zeta potential of LHRss/DOX/TRAIL determined by DLS. (**B**) Particle sizes distribution of LHRss/DOX/TRAIL at N/P ratio of 40 determined by DLS. (**C**) Zeta potential of LHRss/DOX/TRAIL at N/P ratio of 40 determined by DLS. (**D**) TEM images of LHRss/DOX/TRAIL at mass ratio at N/P ratio of 40. (**E**) Agarose gel electrophoresis results (a. agarose gel electrophoresis of LHR/pGL3; b. agarose gel electrophoresis of LHRss/pGL3; c. agarose gel electrophoresis of LHRss/pGL3 at DTT condition). (**F**) Release of DOX from LHRss/DOX/TRAIL.

The zeta potential and particle size of LHRss/DOX/DNA are closely related to the N/P ratio. The LHRss/DOX/pGL3 complexes were prepared at different N/P ratios from 2.5:1 to 80:1. As shown in Figure 2A, all complexes had a positive surface charge with a zeta potential ranging from 10 to 30 mV when the N/P ratio was higher than 10. Figure 2A also shows that the particle size of the LHRss/DOX/pGL3 complexes was less than 200 nm at all the N/P ratios. When the N/P ratio was higher than 10, most LHRss/DOX/pGL3 complexes had a higher zeta potential and a smaller particle size than the low N/P ratio complexes. Among the LHRss, LHRss/DOX/pGL3 was found to have an appropriate size and zeta potential (Figure 2B and 2C) at an N/P ratio at 40, with a particle size and zeta potential of 69 ± 2.14 nm and 30.7 ± 2.94 mV, respectively. TEM showed that the LHRss/DOX/pGL3 complexes formed a compact nanostructure, with a similar size as the results shown in Figure 2D at an N/P of 40. Figure 2E showed that with different N/P ratios from 0.25 to 15, the condensing ability of LHRss was enhanced as the N/P ratio was increased. When the N/P ratio was greater than 5, the pGL3 plasmid was completely condensed, indicating that the compression ability of LHR was improved by cross-linking with the disulfide bond. To confirm this finding, DTT, a reducing agent, was used to break the disulfide bond. As shown in Figure 2E, the LHRss/DOX/pGL3 complexes showed a weaker pGL3 binding affinity in the presence of DTT because of the depolymerization of LHRss. Thus, we presume that the inter-molecular disulfide bond of lipoic acid can be broken by reducing the condition in the cytoplasm, thus reducing the affinity of LHRss for pGL3. The characteristics of the inter-molecular disulfide bond of lipoic acid remain stable extracellularly but not stable in the cytoplasm, which can provide a guarantee for the effective uptake and the effective release of the drug.

### Loading and release at different pHs of DOX

Loading of DOX into LHRss/DOX/TRAIL was performed by Dialysis method. It was found that the DLC was 12.5%wt, and the DLE was as high as 71.4%. DOX released from the micelles was pH-sensitive, as shown by the experiments carried out under two different pH conditions (pH 5.5 and 7.4) (Figure 2F). Through the spontaneous fluorescence behavior of DOX, the content of DOX can be detected. The drug release behavior was detected by dialysis. Thus, if DOX was completely condensed in the LHRss, only the released DOX could be detected. The cumulative release rate increased with the lower pH of the release media, from 54.5% in pH 7.4 medium to 95.5% with 60 h in the media with pH of 5.5, which is most likely due to the release of the drug from the disulfide bond under acidic conditions. It was reported that the glutathione concentration in the cytosol of tumor cells was 100–1,000 times that of normal cells [18]. Kim et al. [19] reported that the disulfide bond could be cleaved rapidly via thiol-disulfide exchange reactions with intracellular reducing molecules, especially glutathione. Our study showed that the prepared nanomicelle could rapidly release drugs under the reducing conditions of tumor cells.

### Cellular uptake

**Figure 3:**
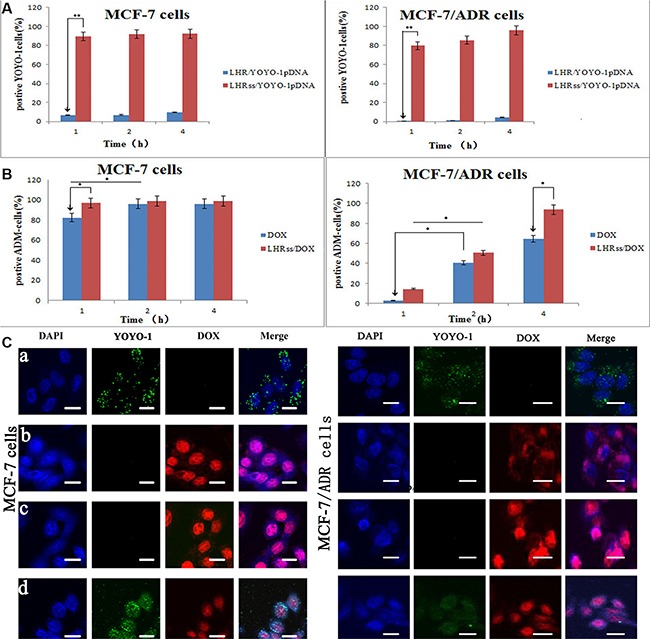
Cellular uptake of DOX and YOYO-1 pDNA (**A**) Quantitative analysis uptake of YOYO-1 pDNA in MCF-7 cells and MCF-7/ADR cells after treated with LHR/YOYO-1 pDNA and LHRss/YOYO-1 pDNA for 1, 2 and 4 h. (**B**) Quantitative analysis of DOX uptake in MCF-7 cells and MCF-7/ADR cells after treated with DOX, LHRss/DOX for 1, 2 and 4 h. (**C**) Confocal microscopic images of MCF-7 cells and MCF-7/ADR cells incubated with LHRss/YOYO-1 pDNA (a), DOX (b), LHRss/DOX (c) and LHRss/DOX/YOYO-1 pDNA (d).

Cellular uptake is an important factor in drug delivery. To determine the cellular uptake of LHRss and the cellular DNA uptake mediated by LHRss/pDNA, the LHR/pDNA in MCF-7 cells and MCF-7/ADR cells was measured using YOYO-1 labeled pDNA. The positive cells were quantitatively assessed by flow cytometry after being incubated for 1, 2, and 4 h. As shown in Figure 3A, the fluorescent signal could not be detected in cells treated with LHR/ YOYO-1pDNA, even after being incubated for 4 h, probably because of the low positive charge of the micelles compressing the DNA and its low affinity to the cell membrane. The cellular uptake of LHRss/ YOYO-1pDNA by MCF-7 cells and MCF-7/ADR cells was time-independent. The uptake of pDNA by MCF-7/ADR cells was slightly more than the uptake by MCF-7 cells. The cellular uptake results showed that the cellular uptake efficiency of the LHRss/pDNA nanomicelle was significantly higher than that of the LHR/pDNA in both MCF-7 and MCF-7/ADR cells. The amount of positive cells was approximately 9.59 fold and 21.05 fold that of the LHR/pDNA nanomicelle in MCF-7 and MCF-7/ADR cells, respectively, probably because cross-linking increased the affinity of the nanocarrier with the cell membrane [20].

Figure 3B shows the uptake of free DOX and LHRss/DOX by MCF-7 and MCF-7/ADR cells. In MCF-7 cells, the cellular uptake of free DOX and LHRss /DOX was both high, and almost saturated at 2 h. However, the uptake of free DOX in MCF-7/ADR cells was low, while the LHRss/DOX had a high accumulation after 4-h treatment, indicating that the uptake might be derived from caveolae endocytosis or micropinocytosis [21]. The high expression of p-gp in the drug resistant cells would efflux DOX, which entered the membrane through electrostatic interaction [22]. However, the micelles could enter the cells through clathrin- or caveolin-mediated endocytosis and bypass the drug efflux pumps, thus increasing the cellular uptake.

To observe the intracellular location of LHRss/YOYO-1pDNA, DOX, LHRss/DOX, and LHRss/DOX/YOYO-1pDNA, DAPI (4′,6-diamidino-2-phenylindole) was used to stain the nucleus. Figure 3Ca shows the intracellular distribution of the micelle in MCF-7 and MCF-7/ADR cells 4 h after transfection. The confocal microscopic images of MCF-7 and MCF-7/ADR cells showed a large amount of green fluorescence in the cytoplasm and a small punctate in the nucleus, which coincided with blue fluorescence, indicating that YOYO-1-labeled pDNA successfully escaped from the endosome and entered the nucleus [23, 24]. These results showed that the LHRss could promote endosomal escape. Figure 3Cb and Figure 3Cc showed the intracellular location of free DOX and LHRss/DOX in MCF-7 and MCF-7/ADR cells. The location of free DOX was basically the same as the LHRss/DOX, indicating that the drug DOX could enter the nucleus to play the cytotoxic effect. While in MCF-7/ADR cells, only a small amount of free DOX was distributed in the cytoplasm and could not enter the nucleus. Interestingly, when MCF-7/ADR cells were treated with LHRss/DOX, the distribution of red fluorescence in the nucleus DOX was significantly increased. The result is consistent with the above cellular uptake. Figure 3Cd showed the co-delivery system of DOX and pDNA, where the green fluorescence was distributed in both cytoplasm and nucleus in both MCF-7 and MCF-7/ADR cells. The red fluorescence was distributed in the nucleus in both MCF-7 cells and MCF-7/ADR cells. These results suggested that the co-delivery system could carry the gene drug and chemotherapy to the cell nucleus.

### Gene transfection efficiency

**Figure 4:**
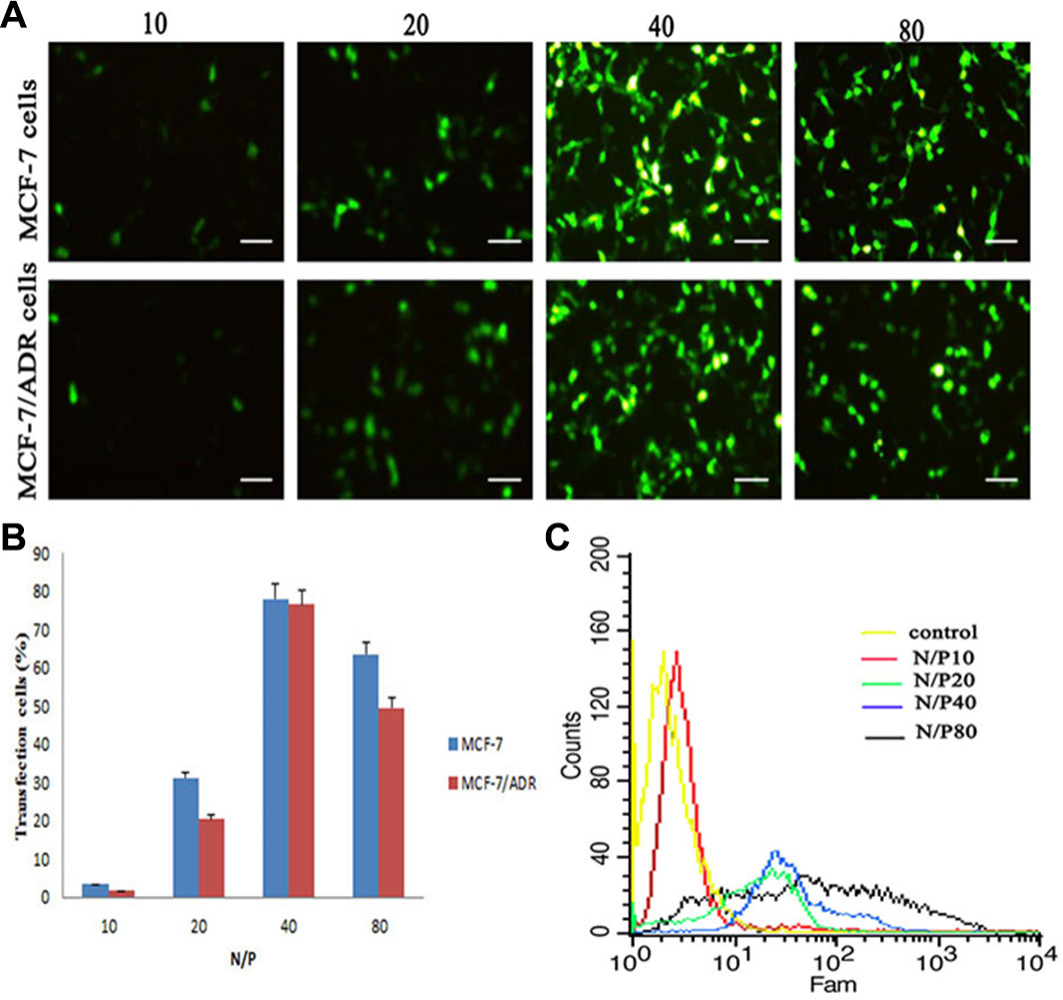
*In vitro* transfection efficiency in MCF-7 cells and MCF-7/ADR cells after treated with LHRss/pEGFP for 48 h (**A**) Microscopic images of MCF-7 cells and MCF-7/ADR cells treated with LHRss/pEGFP at different N/P ratio (10, 20, 40 and 80) for 48 h. (**B**) Quantitative analysis of EGFP expression in MCF-7 cells and MCF-7/ADR cells transfected with LHRss/pEGFP. (**C**) Flow cytometry figures display EGFP expression of MCF-7/ADR cells after treatment with LHRss/pEGFP at different N/P ratio.

The gene transfection efficiency of LHRss was measured using a reporter genes (EGFP) in MCF-7 and MCF-7/ADR cells. It seemed that the gene transfection efficiency was dependent upon the N/P ratio, and this result was observed for both MCF-7 and MCF-7/ADR cells (Figure 4A). The gene transfection efficiency of the LHRss in both MCF-7 and MCF-7/ADR cells at N/P ratio 40 was significantly higher than that in the groups at N/P ratio 20 (*p* < 0.05) (Figure 4B and 4C), most probably because of the enhanced gene compression of the LHRss [25]. It is noteworthy that LHRss showed the optimal gene transfection efficiency at an N/P ratio of 40.

### Cytotoxicity assay

**Figure 5:**
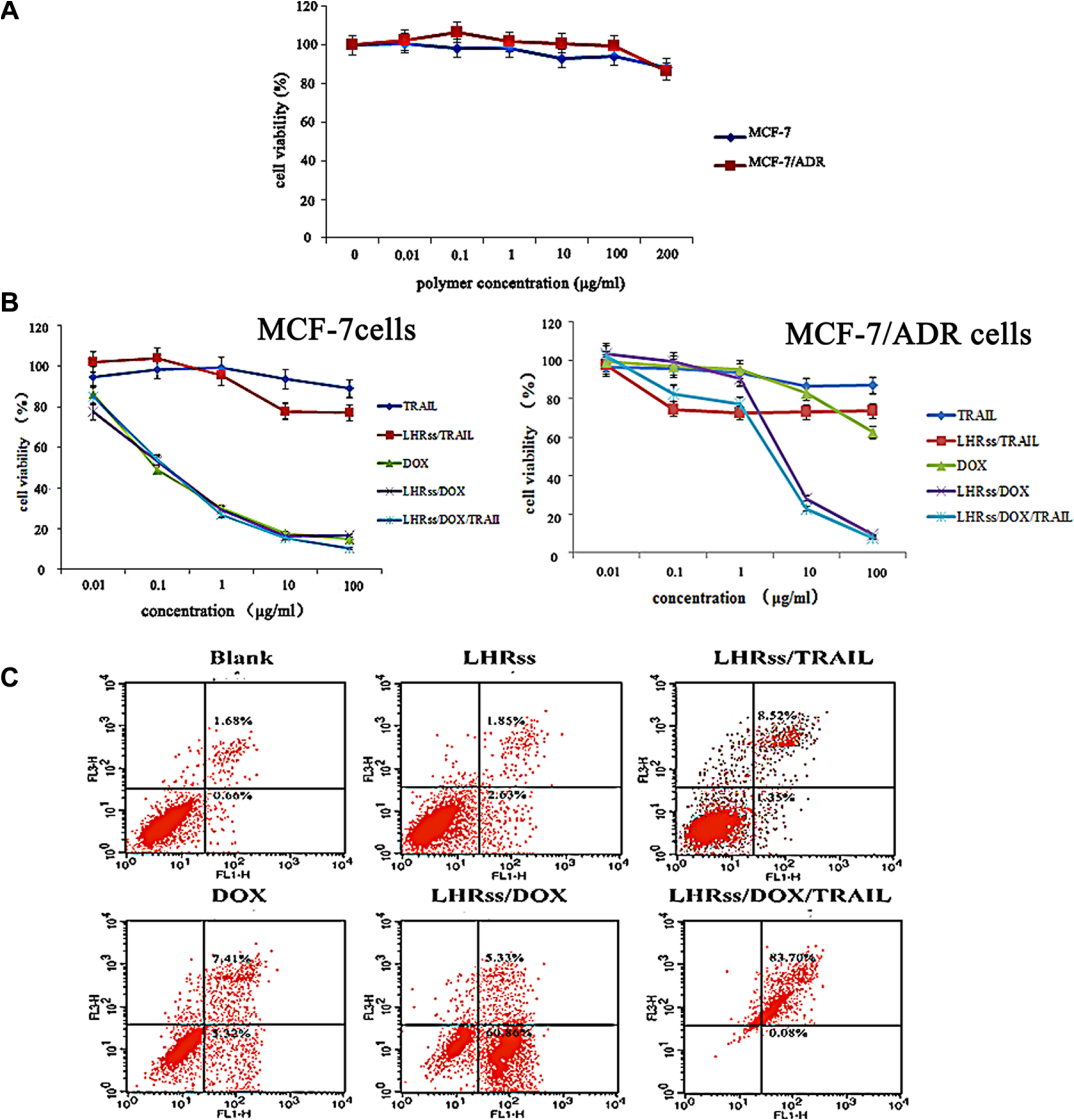
*In vitro* anti-tumor effect of LHRss/DOX/TRAIL (**A**) Viability of MCF-7 cells and MCF-7/ADR cells after treatment with BMPs at different concentrations for 48 h. (**B**) Viability of MCF-7 cells and MCF-7/ADR cells after treatment with TRAIL, LHRss/TRAIL, DOX, LHRss/DOX and LHRss/DOX/TRAIL at different DOX concentrations for 48 h. (**C**) Flow cytometry analysis for apoptosis of MCF-7/ADR cells induced by LHRss, LHRss/TRAIL, DOX, LHRss/DOX and LHRss/DOX/TRAIL.

A successful co-delivery system requires the minimal toxicity of the blank polymer. Figure 5A showed the cytotoxicity of LHRss. The viability of MCF-7 and MCF-7/ADR cells was not significantly affected at the concentrations of LHRss up to 200 μg/mL, indicating that LHRss almost has no cytotoxicity and has a good biocompatibility.

The IC_50_ value of free DOX in MCF-7 and MCF-7/ADR cells at 48 h determined by CCK-8 assay was 2.2 × 10^−4^ and 0.97 mg/mL, respectively, indicating a strong resistance in MCF-7/ADR cells (Figure 5B). LHRss/DOX and LHRss/DOX/TRAIL showed no higher cytotoxicity as compared with free DOX in MCF-7 cells. In MCF-7/ADR cells, LHRss/DOX had an IC_50_ value of 6 × 10^−3^ mg/mL, indicating an increased cytotoxicity by 49.1 fold, which may result from TRAIL and the burst release of DOX induced by the acidic pH of endosomes/lysosomes. The most remarkable killing effect in MDR cells was achieved by LHRss/DOX/TRAIL. The synergy between DOX and TRAIL decreased the IC_50_ value to 2.50 × 10^−3^ mg/mL. In addition, LHRss accelerated the intracellular DOX release and improved the endo/lysosomal escape and the release of TRAIL, making the LHRss/DOX/TRAIL an efficient co-delivery system with good synergy between the drugs and DNA, and caused a significantly decline in MDR *in vitro*.

### Cell apoptosis

Figure 5C showed the apoptosis of MCF-7/ADR cells with each treatment group. MCF-7/ADR cells exposed to BMPs did not show any visible apoptosis after 48-h incubation, which is consistent with the result of CCK-8 assay. Likewise, only 12.73% of the cells were apoptotic because of treatment with free DOX, which failed to afford clear therapy efficacy due to the efflux effect of P-glycoprotein (p-gp). There was no obvious therapeutic effect of the single delivery of pTRAIL, distinctly disclosing the synergistic effect on apoptosis induction in the cells. As shown by the flow cytometry images, LHRss/DOX induced 60.19% cellular apoptosis by benefiting from the up-regulation of TRAIL by LHRss/TRAIL and the intracellular accumulation of DOX. Nevertheless, LHRss/DOX/TRAIL caused 83.78% cell apoptosis via the synergist effect of DOX and TRAIL.

### Biodistribution

**Figure 6:**
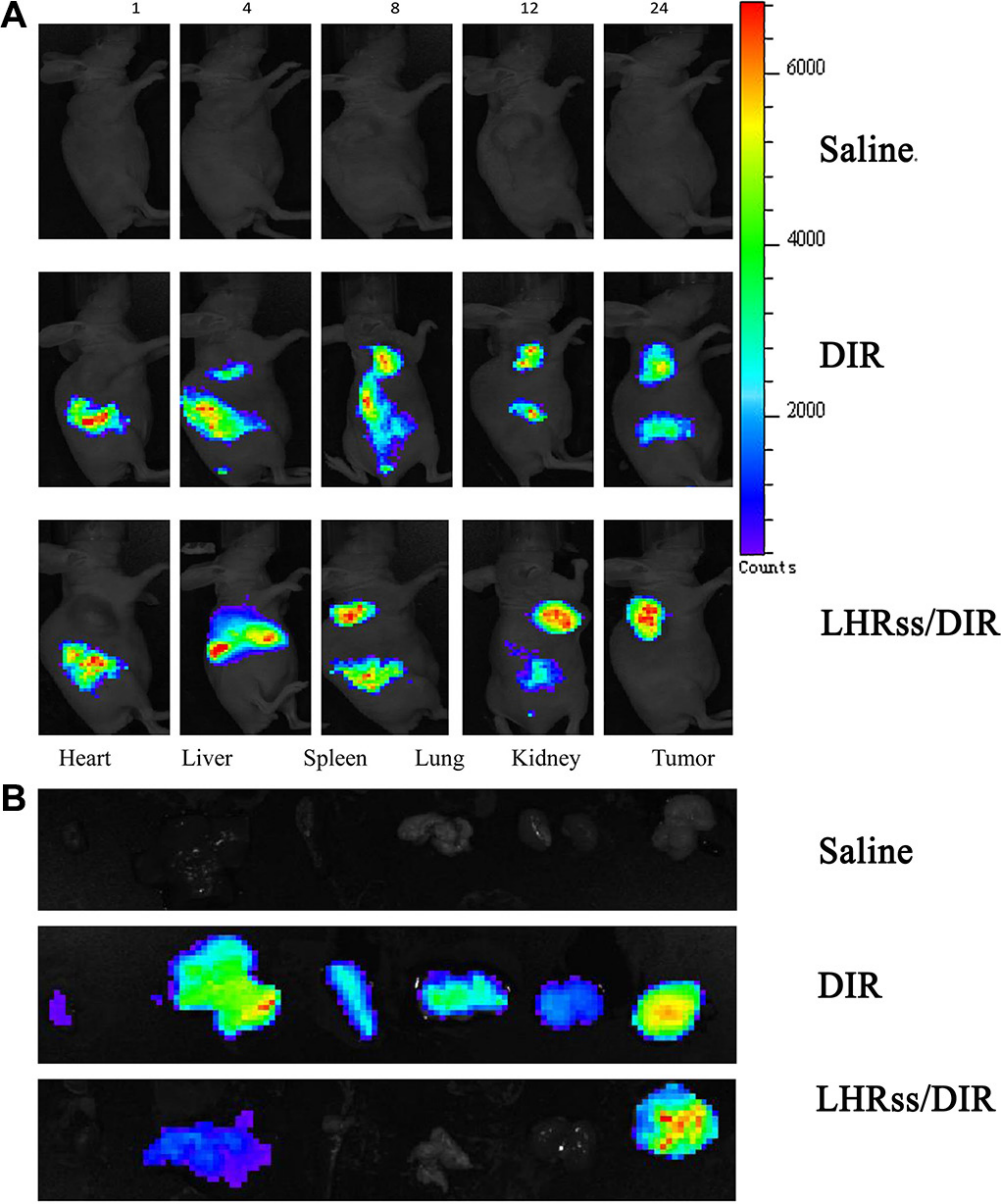
Biodistribution of LHRss/DIR (**A**) Biodistribution of DIR in MCF-7/ADR tumor-bearing mice at 1, 4, 8, 12 and 24 h after intravenous administration of DIR and LHRss/DIR at the dose of 5 mg/kg. (**B**) *Ex vivo* fluorescence images of tissue including heart, liver, spleen, lung, kidneys, and tumor at 24 h after intravenous administration of saline, DIR and LHRss/DIR.

DIR, a lipophilic fluorescent dye, is a model drug for targeted research of nanomicelles [26, 27]. The distribution of free DIR in each tissue was observed using an *in vivo* imaging system. As seen in Figure 6, most free DIR was highly aggregated in the liver and lung, allowing for capture by the reticuloendothelial system (RES) [28]. Thus, the accumulation of free DIR in the tumor was quite low. LHRss/DIR accumulation at the tumor site was increased markedly, while DIR accumulation in the lung, spleen and other organs was decreased substantially. These results indicated that DIR was protected by the nanomicelle due to the enhanced permeability and retention effect [29, 30].

### *In vivo* anti-tumor effect

**Figure 7:**
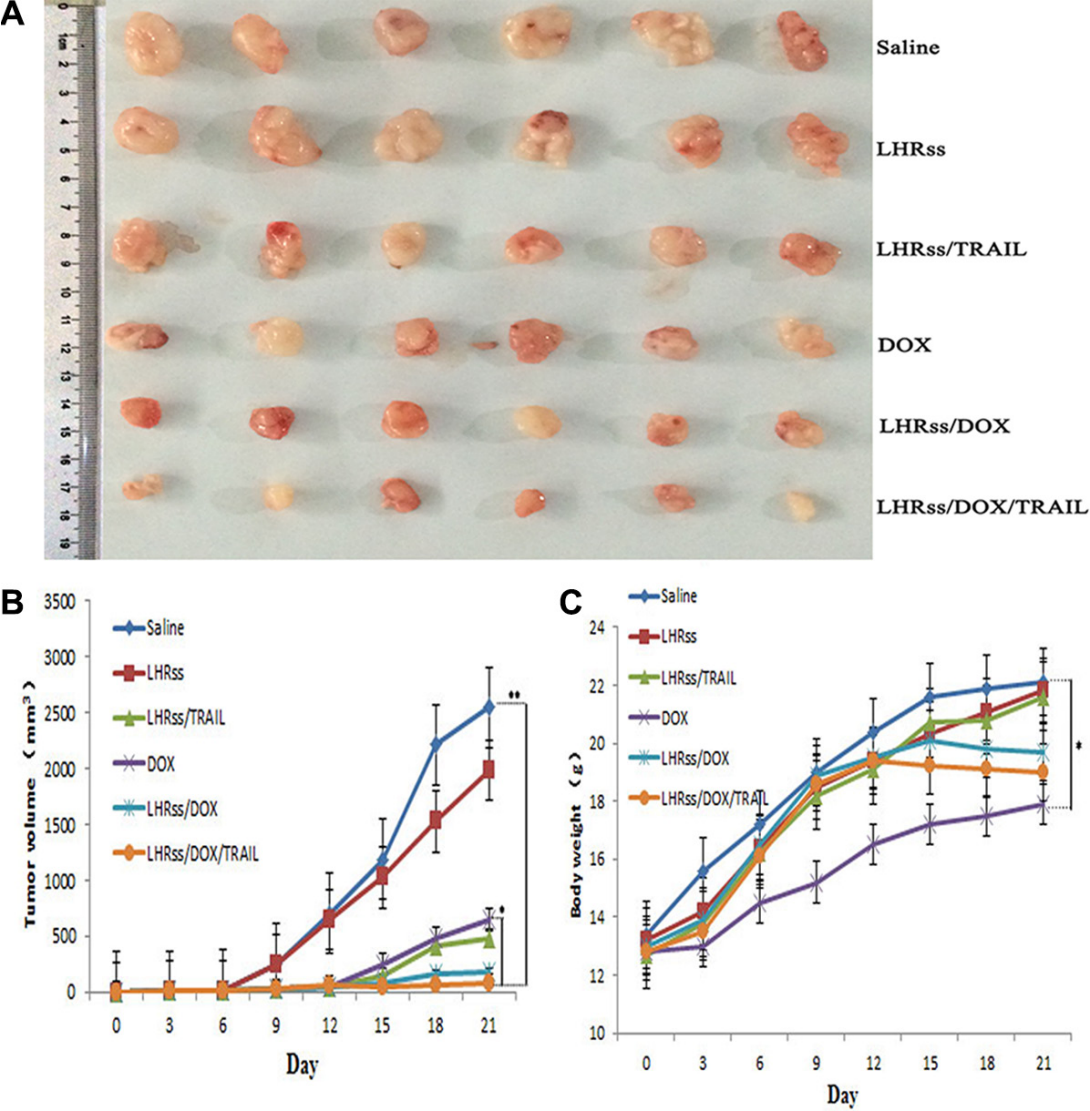
*In vivo* anti-tumor effect (**A**) The picture of the tumors on day 21. (**B**) The tumor inhibiting rate at day 21. (**C**) Body weights of tumor-bearing mice. Date was given as mean ± SD (*n* = 6). (**p < 0.01).

The therapeutic effectiveness of LHRss/DOX/TRAIL was evaluated in MCF-7/ADR tumor-bearing nude mice. Tumor growth in the saline group was used as a control, where the growth rate was so fast that the mean tumor volume increased from 52.1 mm^3^ on the first day to 2,544.6 mm^3^ within 21 days (Figure 7B). The similar result was also observed in BMPs-treatment group, indicating that the nanocarrier itself hardly had any physiological activity. The tumor-inhibitory capacity of LHRss/TRAIL and free DOX was 60.9% and 58.6% TIR, respectively, demonstrating that pDNA could promote tumor apoptosis *in vivo*. LHRss/DOX showed an increased tumor-inhibitory rate of 86.7%, probably due to drug accumulation at the tumor site [33]. The most significant effect was concerned with LHRss/DOX/TRAIL, showing a tumor-inhibitory rate of 94.0%. The final tumor volume was 99.2 mm^3^, which was only 3.9% of the tumor volume in the saline group, and 8.6-fold smaller than that in free DOX group. The predominant therapeutic effect could be triggered by targeted accumulation in the tumor, and the combination with TRAIL promoted apoptosis, which enhanced the sensitivity of tumor cells to chemotherapy [31, 32].

**Figure 8:**
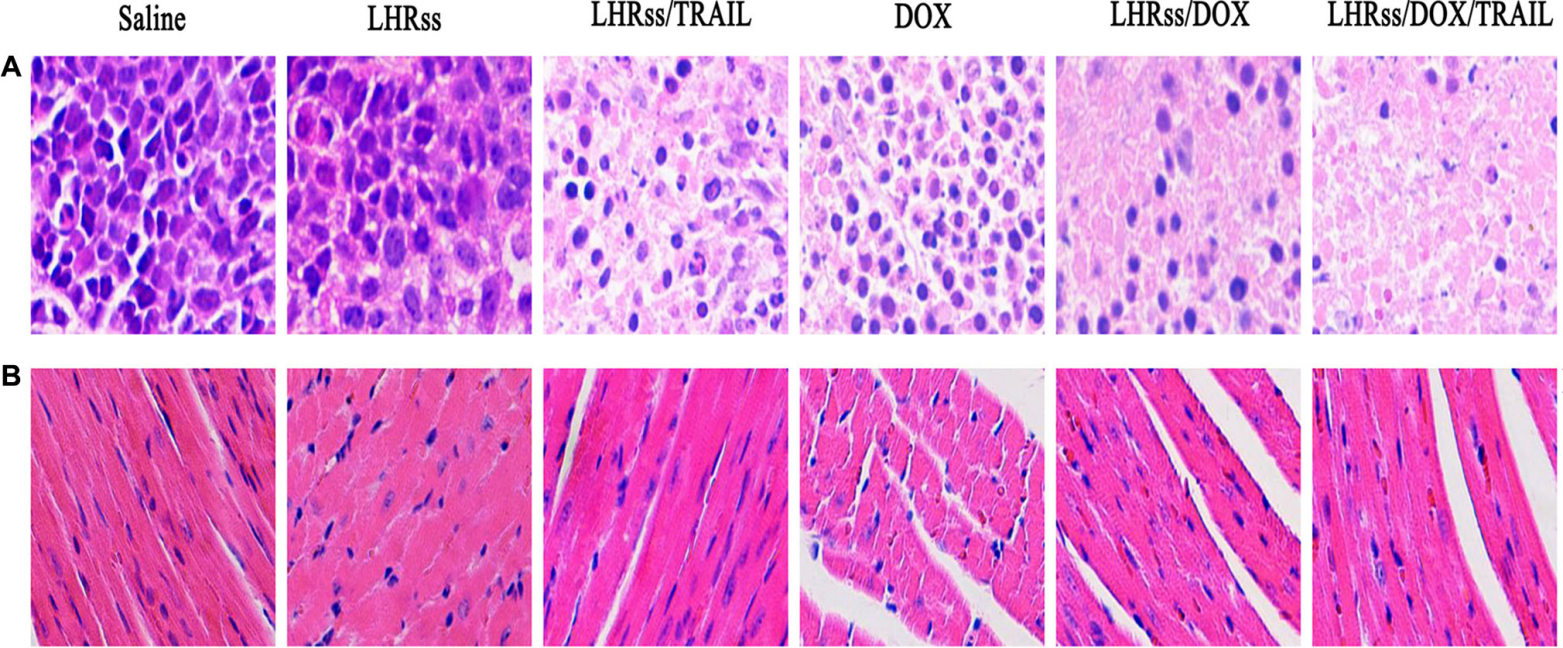
The histological characteristic of MCF-7/ADR tumor tissue and organ histology (**A**) The histological characteristic of MCF-7/ADR tumor tissue after treatment with saline, LHRss, LHRss/TRAIL, DOX, LHRss/DOX, and LHRss/DOX/TRAIL. (**B**) Representative organ histology of saline, LHRss, LHRss/TRAIL, DOX, LHRss/DOX, and LHRss/DOX/TRAIL injected mice.

Body weight change was used an indicator of systemic toxicity (Figure 7C) [34, 35]. Importantly, mice treated with BMPs and LHRss/TRAIL had similar body weight to the saline group, indicating that the nanocarrier had nearly no systemic toxicity. Remarkably, the LHRss/DOX and LHRss/DOX/TRAIL groups had reduced body weight after 15-day treatment, which can be explained by a loss in tumor weight. In contrast, the body weight of free DOX group was decreased substantially, indicating a severe adverse effect. Histological analysis using HE staining showed that treatment with LHRss/DOX/TRAIL resulted in extensive necrosis of the tumor tissue with little damage to the heart. However, free DOX caused significant heart necrosis but little tumor necrosis. In addition, free DOX even led to myocardial atrophy (Figure 8).

**Figure 9:**
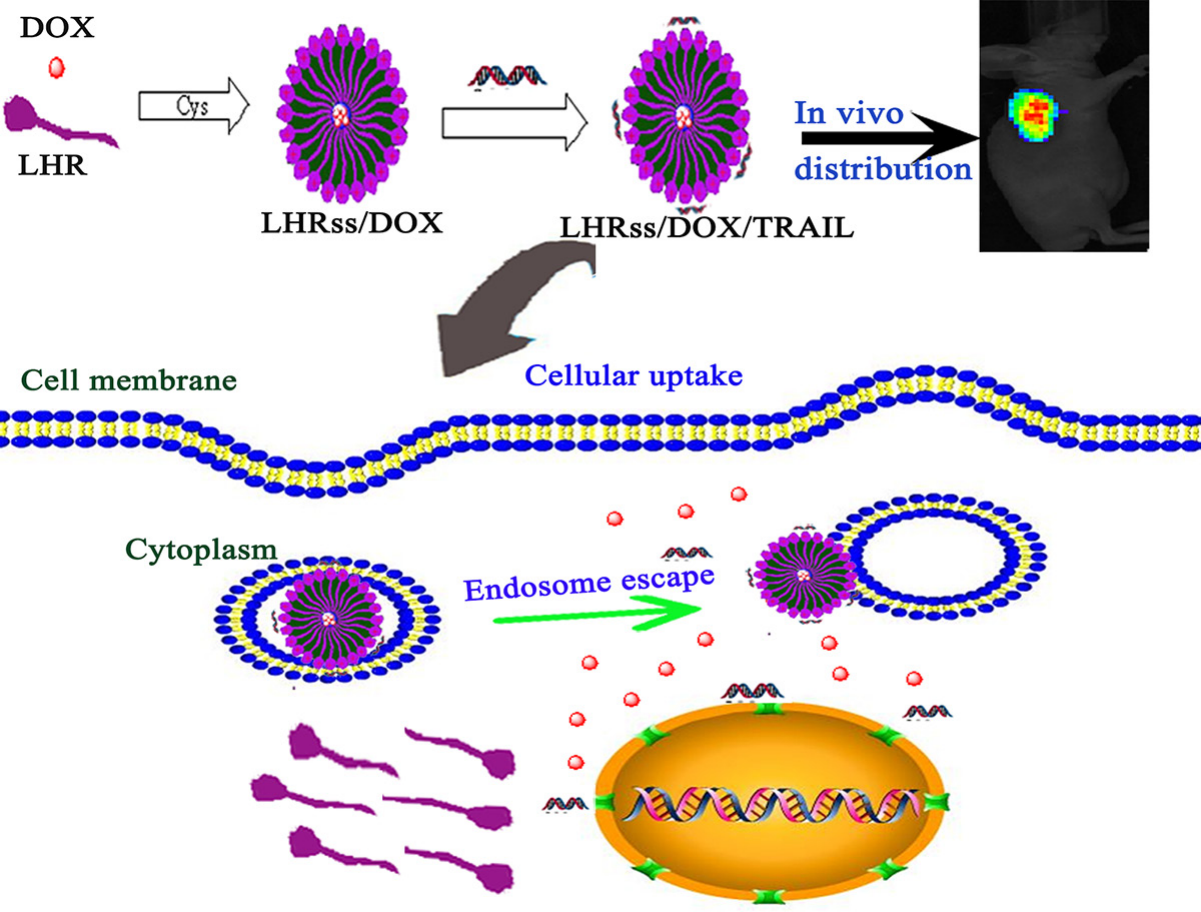
Scheme l The formation of LHRss/DOX/TRAIL and the *in vitro*/*in vivo* effect.

## CONCLUSIONS

In this study, we designed and developed a co-delivery system of DOX and pTRAIL based on LHRss. Due to the intermolecular disulfide bond of lipoic acid, the nanomicelle showed a pH-sensitive behavior, which promoted endosomal escape. LHRss/DOX/TRAIL enhanced the intracellular accumulation of DOX, promoted the expression of TRAIL, and promoted the apoptosis of drug-resistant breast cancer cells. Because of its good biocompatibility, biodegradation and high cytotoxicity, the co-delivery system of LHRss/DOX/TRAIL is expected to be a promising approach for the treatment of drug-resistant breast cancer.

## MATERIALS AND METHODS

### Materials

L-histidine hydrochloride, L-arginine and lipoic acid were purchased from Sangon Biotech (Shanghai, China). L-cysteine hydrochloride monohydrate and dithiothreitol (DTT) were purchased from Sigma-Aldrich (St Louis, MO, USA). A luciferase assay kit was gifted from Promega (Madison, WI, USA). An enhanced bicinchoninic acid protein assay kit was purchased from Beyotime (Nanjing, China). Dulbecco's Modified Eagle' Medium (DMEM), fetal bovine serum (FBS), YOYO-(Y3601) and a penicillin-streptomycin solution (5 KU/mL) were purchased from Life Technologies (Carlsbad, CA, USA). The Cell Counting Kit-8 (CCK-8) was purchased from Dojindo Molecular Technologies, Inc. (Nanjing, China). Propidium iodide (PI) and an apoptosis kit were purchased from Invitrogen (Oregon, USA). pDNA (pGL3, pEGFP and pTRAIL) was purchased from Shanghai Innovation Biotechnology Co., Ltd (Shanghai, China). Doxorubicin (DOX) hydrochloride was gifted from Hisun Pharmaceutical Co., Ltd (Zhejing, China).

### Cell lines and cell culture

The human breast adenocarcinoma MCF-7 cell line was obtained from the American Type Culture Collection (ATCC, Manassas, VA) and grown in DMEM supplemented with 10% FBS and antibiotics (100 U/mL penicillin and streptomycin). The DOX-resistant cell line (MCF-7/ADR cells) was purchased from Gefan Biotech, Co., Ltd (Shanghai, China) and cultured in complete DMEM with 1 μg/mL DOX. Both cell lines were maintained at 37°C in a humidified 5% CO_2_ atmosphere.

### Animals

Male BALB/c nude mice (12–14 g) were purchased from the Department of Experimental Animals of the Second Military Medical University (Shanghai, China). All animal procedures were performed under the guidelines approved by the Institutional Animal Care and Use Committee (IACUC) of the Shanghai Institute of Materia Medica of the Chinese Academy of Sciences.

### Synthesis and characterization of polymers

First, a histidine-arginine peptide (H_3_R_6_, HR) was synthesized using the method of F-moc-solid phase peptide synthesis (SPPS). Second, lipoic acid was coupled to the N-terminus of the HR peptide to obtain lipoic acid HR (LHR) using the same synthesis method. The products were purified by reverse HPLC. Then, the LHR (10 mg) was dissolved in 1 mL methanol, and a methanol solution of L-cysteine hydrochloride was added to the mixture under stirring to serve as a cross-linked catalyst at room temperature for 12 h. Finally, the solvent was removed by N_2_ drying, and the remaining cysteine was neutralized with NaOH. The solution was then lyophilized to obtain LHRss (Figure 1A). The molecular weight of LHR and LHRss was determined by HLC-8220 gel permeation chromatography (TOSOH Corporation, Tokyo, Japan). The synthesized polymers were also characterized by ^1^H-NMR at 600 MHz (Varian Inc., Palo Alto, CA, USA) in deuterium oxide.

### Preparation and characterization of LHRss/DOX/TRAIL micelle

#### Co-delivery of pDNA and DOX by LHRss

pGL3 was used as a model plasmid. For the DOX-entrapped LHRss (LHRss/DOX), 2 mg DOX·HCl was dissolved in 1 mL acetonitrile, and 5 μl trimethylamine was added to the solution to transform DOX·HCl into hydrophobic DOX. Then, the DOX solution and sodium cholate hydrate (1%) were placed into the LHRss solution. The organic solution was then mixed with 20 mL distilled water and vigorously ultrasonicated. The solvent was evaporated by vigorous stirring at 1,000 rpm overnight to remove the acetonitrile. The resulting nanomicelle suspension was centrifuged at 10,000 rpm for 15 min. The supernatant then was passed through a 0.220 μm syringe filter to eliminate the polymer and DOX aggregates. The LHRss/(DOX/pDNA) was prepared by mixing the pDNA solution (20 μM) with a specified amount of the LHRss/DOX solution, and the mixture was incubated at room temperature for 30 min to electrostatically bind the negative pDNA to the positive surface of the nanocarrier. All procedures were carried out in the dark.

#### Size and zeta potential of the complex

pGL3 was used as a model plasmid. The LHRss/DOX complex was mixed with pGL3 at different N/P ratios in 1 mL PBS (19 mM, pH 7.4), and the mixture was incubated at room temperature for 30 min. Then, the particle size and zeta potential of the complex were further determined using dynamic light scattering (Zetasizer Nano ZS90, Malvern Instruments, Malvern, USA). The morphology of the LHRss/pGL3 complex at an N/P ratio of 40 was examined under a transmission electron microscope (Hitachi, Tokyo, Japan) at an acceleration voltage of 75 kV.

#### Drug loading efficiency and drug release

The loading efficiency of DOX within the nanocarrier was measured using a GloMax-Multi Jr Single Tube Multimode Reader (Promega). Briefly, 5 mg nanocarrier was dissolved in 1 ml PBS (pH 7.4) using vigorous vortexing. This solution was transferred to a 200-μl capillary tube. The measurement was performed in triplicate. For the determination of the drug loading content (DLC) and the drug loading efficiency (DLE), lyophilized drug-loaded nanoparticles were dissolved in DMSO. The amount of DOX was determined using fluorescence measurements. The DLC and DLE were calculated according to the following formulas:

DLC (wt%) = (wt of loaded drug/total wt of polymer and loaded drug)×100%

DLE (%) = (weight of loaded drug/weight of drug in feed)×100%

The pH-dependence of the drug release behavior of the DOX-LHRss nanocarrier was shown using a GloMax-Multi Jr Single Tube Multimode Reader. To examine the pH-dependent dye release, DOX-loaded nanocarrier solutions (10%) were treated with solutions of disodium hydrogen phosphate citrate buffer at different pHs (pH 5.5 and 7.4). The amount of DOX released at each time point was determined by fluorescence detector analysis.

#### Agarose gel electrophoresis

The condensation ability of the complexes was determined by agarose gel electrophoresis. The complexes were prepared at different N/P ratios (0.25–15). After they were incubated for 30 min, the complexes, which included 1 μg pGL3, were added to the pores of an acetic acid-EDTA buffer and TAE-containing 1% agarose gel. The gel was run at 100 V for 30 min. The nucleic acid framework was irradiated under UV.

The DNA release ability was evaluated by salt separation. Complexes with an N/P ratio of 20 were prepared and incubated in 25 nM DTT at 37°C for 2 h. Samples were analyzed with agarose gel electrophoresis under the same conditions.

#### Cellular uptake of YOYO-1-DNA and intracellular accumulation of DOX

The DNA uptake by MCF-7 cells and MCF-7/ADR cells was analyzed using flow cytometry. MCF-7 cells and MCF-7/ADR cells were seeded into 12-well plates at 2 × 10^5^ cells per well and incubated for 24 h. After replacing the culture medium, the LHRss/YOYO-1pDNA or with the DOX solution and the LHRss/DOX were added to the MCF-7 and MCF-7/ADR cells, with a final YOYO-1pDNA concentration of 75 nM, and a final DOX concentration of 5 μg/mL. After they were incubated for 1, 2, and 4 h, the cells were washed, trypsinized, centrifuged, and re-suspended in 300 μl PBS. The cells were analyzed on a FACScan flow cytometer (Becton Dickinson, San Jose, CA, USA).

### CLSM observation

MCF-7 cells and MCF-7/ADR cells were seeded into glass-bottom 24-well plates at a density of 1 × 10^5^ cells per well and incubated for 24 h. After replacing the culture medium, the LHRss/ YOYO-1pDNA with or without free DOX solution, LHRss/DOX, or LHRss/ DOX/YOYO-1pDNA were added to MCF-7 cells and MCF-7/ADR cells with a final YOYO-1pDNA concentration of 75 nM and a final DOX concentration of 5 μg/mL. After incubation for 1, 2, and 4 h, the medium was replaced, and the culture was expanded. At different times after transfection, the cells were fixed using 4% paraformaldehyde and treated with 4,6-diamidino-2-phenylinole dihydrochloride to stain the nucleus. Then, the cells were washed, sealed with mounting medium, and imaged using a CLSM.

### Gene transfection assay

The *in vitro* gene transfection efficacy of the LHRss/DNA complexes was evaluated using MCF-7 cells and MCF-7/ADR cells. pEGFP was used as the reporter gene to compare the transfection efficiency of the LHRss-based gene carrier. Transfection experiments were performed using 24-well plates. MCF-7 cells and MCF-7/ADR cells were seeded into 24-well plates at 2 × 10^5^ cells per well and incubated for 24 h. Before transfection, the serum-free medium was replaced. Complexes including 1 μg pDNA (pEGFP) were placed into each plate with different N/P ratios. After 4-h incubation, the culture medium was replaced with 10% FBS. For pEGFP transfection, the gene expression was detected by fluorescence microscopy (Leica, Germany). The expression of EGFP was analyzed by flow cytometry. Forty-eight hours after transfection, the cells were digested and suspended in 300 μl PBS. The fluorescence intensity was detected by flow cytometry (BD, USA).

### Cytotoxicity assay

To evaluate the cytotoxicity of LHRss, a CCK-8 assay was performed. Briefly, MCF-7 and MCF/ADR cells were seeded at a density of 1 × 10^4^ cells per well in 96-well plates for 24 h. The medium was then replaced with fresh culture medium containing various concentrations of the polymer. Cells without treatment were used as the control. The final concentrations of the polymer ranged from 1 × 10^−5^ −2 × 10^−1^ mg/mL. After 24 h incubation, fresh medium containing a 10% CCK-8 solution was added. The absorbance of each well was measured at 450 nm using a microplate reader (Thermo Fisher Scientific, Waltham, MA, USA). The absorbance of the untreated cells was set at 100%, and the cell viability was expressed as the percentage relative to the absorbance of the untreated cells.

The cytotoxicity of the polymers for MCF-7 cells and MCF-7/ADR cells was evaluated with a CCK-8 assay. MCF-7 cells and MCF-7/ADR cells were inoculated into 96 well-plates with densities of 8 × 10^3^ cells per well and incubated for 24 h. Then, different concentrations of DOX, LHRss/DOX, LHRss/TRAIL and LHRss/ DOX/TRAIL were added to each well. After 24-h incubation, fresh medium containing a 10% CCK-8 solution was added. The absorbance of each well was measured at 450 nm using a microplate reader (Thermo Fisher Scientific, Waltham, MA, USA). The absorbance of the untreated cells was set at 100%, and cell viability was expressed as the percentage relative to the absorbance of the untreated cells. The experiment was repeated three times.

### Cell apoptosis

To determine the effect of LHRss/DOX/TRAIL on cell apoptosis, MCF-7/ADR cells seeded in 12-well plates (3 × 10^5^ cells/well) were treated with LHRss, DOX, LHRss/TRAIL, LHRss/DOX, and LHRss/TRAIL/DOX (5 μg/mL DOX and 1 μg/mL TRAIL) for 48 h. Cells without treatment were used as control. For the quantitative measurement of apoptosis, cells were harvested, washed twice with ice-cold PBS and then stained with Annexin V-FITC and PI for 15 min at room temperature in the dark. The apoptosis was analyzed by flow cytometry.

### Biodistribution and *in vivo* anti-tumor effect

A subcutaneous tumor model was generated by injection of 1 × 10^6^ MCF-7/ADR cells into the right axilla of nude mice. The tumors were allowed to grow to approximately 100 mm^3^ before the experiment. To determine the tissue distribution of DOX and TRAIL, DIR, a lipophilic fluorescent dye was used as the model drug for the targeted research of nanomicelles. Female nude mice bearing MCF-7/ADR breast cancer were randomly assigned to 3 groups (*n* = 3) and injected with PBS, DIR, LHRss/DIR (DIR of 50 μg/kg) through the tail vein. The mice were sacrificed 24 h later to excise the heart, liver, spleen, lung, kidney and the tumor. The excise organs and tumor were washed with cold saline and imaged using the FX Pro *in vivo* imaging system (Carestream Health, USA).

An *in vivo* anti-tumor effect assay was carried out as follows: mice bearing visible MCF-7/ADR tumors were randomly divided into saline, LHRss, LHRss/TRAIL, DOX, LHRss/DOX, and LHRss/DOX/TRAIL groups (*n* = 6 each group). The mice were intravenously administered the respective formulation once per week at a dose of 6 mg/kg DOX and 2 mg/kg TRAIL. The body weight and tumor volumes ([major axis] × [minor axis] 2/2, measured by calipers) were monitored and recorded twice per week over a period of 21 days. Then, the mice were sacrificed, and their tumors were excised, weighed and photographed. The tumor inhibitory rate (TIR) was calculated using the following equation:

TIR= (1 –Wtest/Wsaline) × 100%

Where W test is the mean tumor weight of the tested groups, and Wsaline refers to the mean tumor weight of the saline group.

### Statistical analysis

All values are presented as the mean ± S.D. Each value is the mean of at least three repetitive experiments in each group. The statistical significance was determined using Student's *t*-test. The differences were considered significant for *p < 0.05 and very significant for **p < 0.01.
